# Supplementary material for: Factors associated with oral anticoagulant non-use at first ischemic stroke in atrial fibrillation: A nationwide study
Source: Eur Stroke J. 2025 Jun 20:23969873251343857. Online ahead of print. doi: 10.1177/23969873251343857 (PMC12182559; doi:10.1177/23969873251343857)

**Supplementary material**

**Table S1. Definition of the Comorbidities.**

|  | ICD-10 | ICPC-2 | Reimbursement code | ATC-code |
| --- | --- | --- | --- | --- |
| Hypertension | I10-I15 | K85  K86  K87 | 205 | C03A, C03B, C03DB, C03EA, C07A, C08CA, C08D, C09 |
| Diabetes | E10-E14 | T89  T90 | 103  215 | A10 |
| Hyperlipidemia | E78 | T93 | 206 | C10 |
| Coronary artery disease | I20-I25, Z95.1 | - | - | - |
| Myocardial infarction | I21-I22 | - | - | - |
| Vascular disease | I65-I66, I672, I70 | - | - | - |
| Congestive heart failure | I50 | K77 | 201 | - |
| Cardiomyopathy | I420-I422, I429 | - | - | - |
| Ischemic stroke  or TIA | I63 (I63.6 excluded), I64  I693-I698  G45 (G45.4 excluded) | K90 | - | - |
| Bleeding history | D50.0, D62, D68.3, I60-I62, I69.0-I69.2, I85.0, I86.4, J94.2, K22.1, K22.3, K22.6, K25.0, K25.2, K25.4, K25.6, K26.0, K26.2, K26.4, K26.6, K27.0, K27.2, K27.4, K27.6, K28.0, K28.2, K28.4, K28.6, K29.0, K62.5, K63.1, K63.3, K92.0-K92.2, N02, R04, R31, R58, S06.2-S06.6, S06.8 |  |  |  |
| Alcohol abuse | F10 |  |  |  |
| Renal failure or dialysis | N18, Z49 |  |  |  |
| Liver cirrhosis or failure | K70.2-K70.4, K71.7, K71.8, K72, K74 |  |  |  |
| Venous thromboembolism | I26, I80-I83 | - | - | - |
| Thyrotoxicosis | E05 | - | - | - |
| Dementia | F00, F01, F02, F03 | - | - | - |
| Psychiatric disease | F04-F99 | - | - | - |
| Antiplatelets/NSAID |  |  |  | M01A, M01B, L01 |

ICD-10 indicates International Classification of Diseases, Tenth Revision; ATC, anatomic therapeutic chemical; ICPC-2, International Classification of Primary Care, Second Edition; NSAID, non-steroidal anti-inflammatory drug; TIA transient ischemic attack.

**Table S2.** Oral anticoagulation (OAC) purchases in non-stroke patients: 120- and 365-day sensitivity analysis

| Covariate | No-stroke patients (n=157 376)  OAC purchases 365 days after  cohort entry | No-stroke patients (n=157 376)  OAC purchases 120 days after  cohort entry |
| --- | --- | --- |
| Age, y |  |  |
| **<65** | **2.596 (2.520–2.675)** | **2.470 (2.397–2.544)** |
| 65-74 | 0.867 (0.843–0.892) | 0.909 (0.884–0.934) |
| ≥75 | Reference | Reference |
| **Female sex** | **1.042 (1.018–1.066)** | **1.068 (1.044–1.092)** |
| Level of education |  |  |
| 1^st^ | 0.839 (0.814–0.866) | 0.822 (0.797–0.847) |
| 2^nd^ | 0.796 (0.771–0.822) | 0.793 (0.768–0.818) |
| 3^rd^ | Reference | Reference |
| Income level |  |  |
| **1^st^** | **1.339 (1.301–1.378)** | **1.291 (1.255–1.328)** |
| **2^nd^** | **1.060 (1.031–1.090)** | **1.041 (1.013–1.069)** |
| 3^rd^ | Reference | Reference |
| Hypertension | 0.731 (0.713–0.749) | 0.772 (0.753–0.791) |
| Diabetes | 0.742 (0.722–0.764) | 0.760 (0.739–0.781) |
| **Vascular disease** | **1.224 (1.158–1.294)** | **1.254 (1.188–1.325)** |
| Hyperlipidemia | 0.663 (0.647–0.679) | 0.687 (0.671–0.703) |
| Congestive heart failure | 0.811 (0.787–0.836) | 0.793 (0.770–0.817) |
| **Chronic kidney disease** | **1.752 (1.655–1.854)** | **1.709 (1.616–1.808)** |
| **Liver dysfunction** | **2.343 (2.007–2.734)** | **2.248 (1.922–2.629)** |
| **Prior bleeding** | **1.514 (1.460–1.570)** | **1.512 (1.458–1.568)** |
| **Alcohol use disorder** | **1.578 (1.479–1.683)** | **1.579 (1.480–1.685)** |
| **Coronary artery disease** | **1.131 (1.092–1.171)** | **1.131 (1.093–1.170)** |
| **Myocardial infarction** | **1.237 (1.179–1.297)** | **1.225 (1.170–1.284)** |
| Cardiomyopathy | 0.583 (0.537–0.634) | 0.622 (0.574–0.674) |
| Venous thromboembolism | 0.787 (0.752–0.823) | 0.804 (0.770–0.841) |
| **Dementia** | **2.517 (2.391–2.651)** | **2.385 (2.264–2.511)** |
| Thyrotoxicosis | 0.903 (0.807–1.010) | 0.891 (0.798–0.994) |
| Psychiatric disease | 0.995 (0.957–1.033) | 0.975 (0.939–1.012) |
| **Antiplatelets/NSAID** | **1.024 (1.000–1.048)** | **1.032 (1.008–1.056)** |

NSAID indicates non-steroidal anti-inflammatory drug.

|  | **Alive within 30 days after first-ever IS**  **(n=9 790)** | **Death within 30 days after first-ever IS**  **(n=1 890)** | **P-value** |
| --- | --- | --- | --- |
| **Demographics** | | | |
| Age at the time of stroke, years | 77.9 (10.6) | 84.9 (8.6) | <0.001 |
| Age group | | | <0.001 |
| <65 | 1 216 (12.4) | 56 (3.0) |  |
| 65-74 | 2 227 (22.7) | 180 (9.5) |  |
| ≥75 | 6 347 (64.8) | 1 654 (87.5) |  |
| Female sex | 5 390 (55.1) | 1 257 (66.5) | <0.001 |
| Level of education |  |  | <0.001 |
| 1^st^ | 5 902 (60.3) | 1 364 (72.2) |  |
| 2^nd^ | 2 294 (23.4) | 305 (16.1) |  |
| 3^rd^ | 1 594 (16.3) | 221 (11.7) |  |
| Income level |  |  | <0.001 |
| 1^st^ | 3 820 (39.0) | 897 (47.5) |  |
| 2^nd^ | 3 080 (31.5) | 557 (29.5) |  |
| 3^rd^ | 2 890 (29.5) | 436 (23.1) |  |
| **Comorbidities** | | | |
| Hypertension | 7 918 (80.9) | 1 583 (83.8) | 0.003 |
| Diabetes | 2 450 (25.0) | 513 (27.1) | 0.053 |
| Vascular disease | 876 (8.9) | 219 (11.6) | <0.001 |
| Hyperlipidemia | 4 859 (49.6) | 789 (41.7) | <0.001 |
| Congestive heart failure | 3 045 (31.1) | 1 022 (54.1) | <0.001 |
| Chronic kidney disease | 496 (5.1) | 161 (8.5) | <0.001 |
| Liver dysfunction | 110 (1.1) | 19 (1.0) | 0.652 |
| Prior bleeding | 2 104 (21.5) | 508 (26.9) | <0.001 |
| Alcohol use disorder | 662 (6.8) | 90 (4.8) | 0.001 |
| Coronary artery disease | 3 543 (36.2) | 824 (43.6) | <0.001 |
| Myocardial infarction | 1 247 (12.7) | 382 (20.2) | <0.001 |
| Cardiomyopathy | 304 (3.1) | 48 (2.5) | 0.188 |
| Venous thromboembolism | 802 (8.2) | 174 (9.2) | 0.145 |
| Dementia | 906 (9.3) | 385 (20.4) | <0.001 |
| Thyrotoxicosis | 141 (1.4) | 26 (1.4) | 0.829 |
| Psychiatric disease | 1 677(17.1) | 319 (16.9) | 0.790 |
| **Risk scores** | | | |
| CHA_2_DS_2_-VA | 3.3 (1.4) | 4.0 (1.2) | <0.001 |
| Modified HAS-BLED | 2.4 (0.9) | 2.5 (0.9) | <0.001 |
| **Use of antithrombotic medication** |  |  |  |
| Anticoagulation status |  |  | <0.001 |
| None | 6 219 (63.5) | 1 288 (68.1) |  |
| Warfarin | 2 943 (30.1) | 509 (26.9) |  |
| DOAC | 628 (6.4) | 93 (4.9) |  |
| Antiplatelets/NSAID | 3 958 (40.4) | 592 (31.3) | <0.001 |

**Table S3.** Comparison of clinical characteristics of atrial fibrillation (AF) patients with first-ever ischemic stroke (n=11 680). Surviving versus not Surviving 30 Days after the Stroke

Data are n (%) or mean (standard deviation). IS indicates ischemic stroke; DOAC, direct oral anticoagulant; CHA_2_DS_2_-VA, stroke risk score in patients with atrial fibrillation according to congestive heart failure, hypertension, age ≥ 75 years, diabetes, stroke or transient ischemic attack (none), vascular disease, and age 65-74 years; Modified HAS-BLED, bleeding risk score in patients with atrial fibrillation according to hypertension, chronic kidney disease, liver dysfunction, prior stroke (none), bleeding history, age ≥ 65 years, concomitant antiplatelets/NSAID use and alcohol (without labile INR); INR, international normalized ratio; NSAID, non-steroidal anti-inflammatory

| Covariate | Model 1  Non-use vs. any OAC    Adjusted odds ratio (95% CI) | Model 2  Non-use vs. Warfarin    Adjusted odds ratio (95% CI) | Model 3  Non-use vs. DOAC    Adjusted odds ratio (95% CI) |
| --- | --- | --- | --- |
| Age, y |  |  |  |
| <65 | 1.535 (0.797–2.954) | 1.408 (0.716–2.770) | 2.907 (0.375–22.551) |
| 65-74 | 0.777 (0.548–1.103) | 0.899 (0.615–1.314) | 0.396 (0.207–0.755) |
| ≥75 | Reference | Reference | Reference |
| **Female sex** | **1.342 (1.070–1.685)** | **1.360 (1.070–1.728)** | 1.251 (0.762–2.052) |
| Level of education |  |  |  |
| **1^st^** | 1.153 (0.823–1.616) | 1.029 (0.715–1.482) | **2.012 (1.078–3.758)** |
| 2^nd^ | 0.966 (0.654–1.426) | 0.911 (0.598–1.387) | 1.313 (0.630–2.736) |
| 3^rd^ | Reference | Reference | Reference |
| Income level |  |  |  |
| **1^st^** | **1.376 (1.043–1.817)** | 1.321 (0.985–1.773) | 1.710 (0.957–3.054) |
| 2^nd^ | 1.085 (0.815–1.445) | 1.035 (0.765–1.401) | 1.416 (0.771–2.600) |
| 3^rd^ | Reference | Reference | Reference |
| Hypertension | 0.851 (0.638–1.135) | 0.888 (0.656–1.201) | 0.584 (0.290–1.175) |
| Diabetes | 0.794 (0.631–0.998) | 0.772 (0.607–0.981) | 0.952 (0.564–1.605) |
| Vascular disease | 0.930 (0.682–1.269) | 0.900 (0.650–1.247) | 1.079 (0.530–2.198) |
| Hyperlipidemia | 0.713 (0.572–0.890) | 0.744 (0.589–0.940) | 0.572 (0.351–0.931) |
| Congestive heart failure | 0.730 (0.593–0.897) | 0.709 (0.570–0.883) | 0.888 (0.566–1.392) |
| **Chronic kidney disease** | **1.875 (1.269–2.768)** | **1.730 (1.154–2.594)** | **3.182 (1.098–9.225)** |
| Liver dysfunction | 1.410 (0.487–4.078) | 1.436 (0.454–4.543) | 1.390 (0.161–12.019) |
| Prior bleeding | 0.957 (0.763–1.200) | 1.135 (0.888–1.451) | 0.429 (0.273–0.673) |
| **Alcohol use disorder** | **1.813 (1.009–3.258)** | 1.750 (0.936–3.272) | 2.277 (0.632–8.201) |
| Coronary artery disease | 1.155 (0.892–1.494) | 1.057 (0.808–1.384) | 2.368 (1.229–4.561) |
| Myocardial infarction | 1.007 (0.746–1.359) | 1.109 (0.809–1.520) | 0.485 (0.236–0.996) |
| Cardiomyopathy | 0.721 (0.393–1.321) | 0.690 (0.367–1.297) | 0.878 (0.231–3.335) |
| Venous thromboembolism | 0.793 (0.567–1.109) | 0.864 (0.604–1.238) | 0.583 (0.302–1.126) |
| **Dementia** | **1.470 (1.131–1.910)** | **1.507 (1.139–1.993)** | 1.277 (0.713–2.285) |
| Thyrotoxicosis | 0.829 (0.358–1.919) | 0.788 (0.330–1.886) | 1.219 (0.150–9.876) |
| Psychiatric disease | 0.905 (0.668–1.227) | 0.912 (0.662–1.256) | 0.841 (0.423–1.675) |
| Antiplatelets/NSAID | 1.103 (0.889–1.369) | 1.096 (0.872–1.379) | 1.107 (0.692–1.772) |

**Table S4.** Logistic regression analysis of independent variables associated with non-use of oral anticoagulation among atrial fibrillation patients with first-ever ischemic stroke. Patients who died within 30 days after stroke (n=1 890).
Comparison of non-use vs. use of oral anticoagulation (OAC) (Model 1), comparison of non-use of oral anticoagulation vs. warfarin use (Model 2) and comparison of non-use of oral anticoagulation vs. direct oral anticoagulation (DOAC) use (Model3).

OR indicates Odds Ratio; CI, Confidence Interval; NSAID, non-steroidal anti-inflammatory drug.

**Figure S1. Flowchart showing inclusion and exclusion criteria**

**
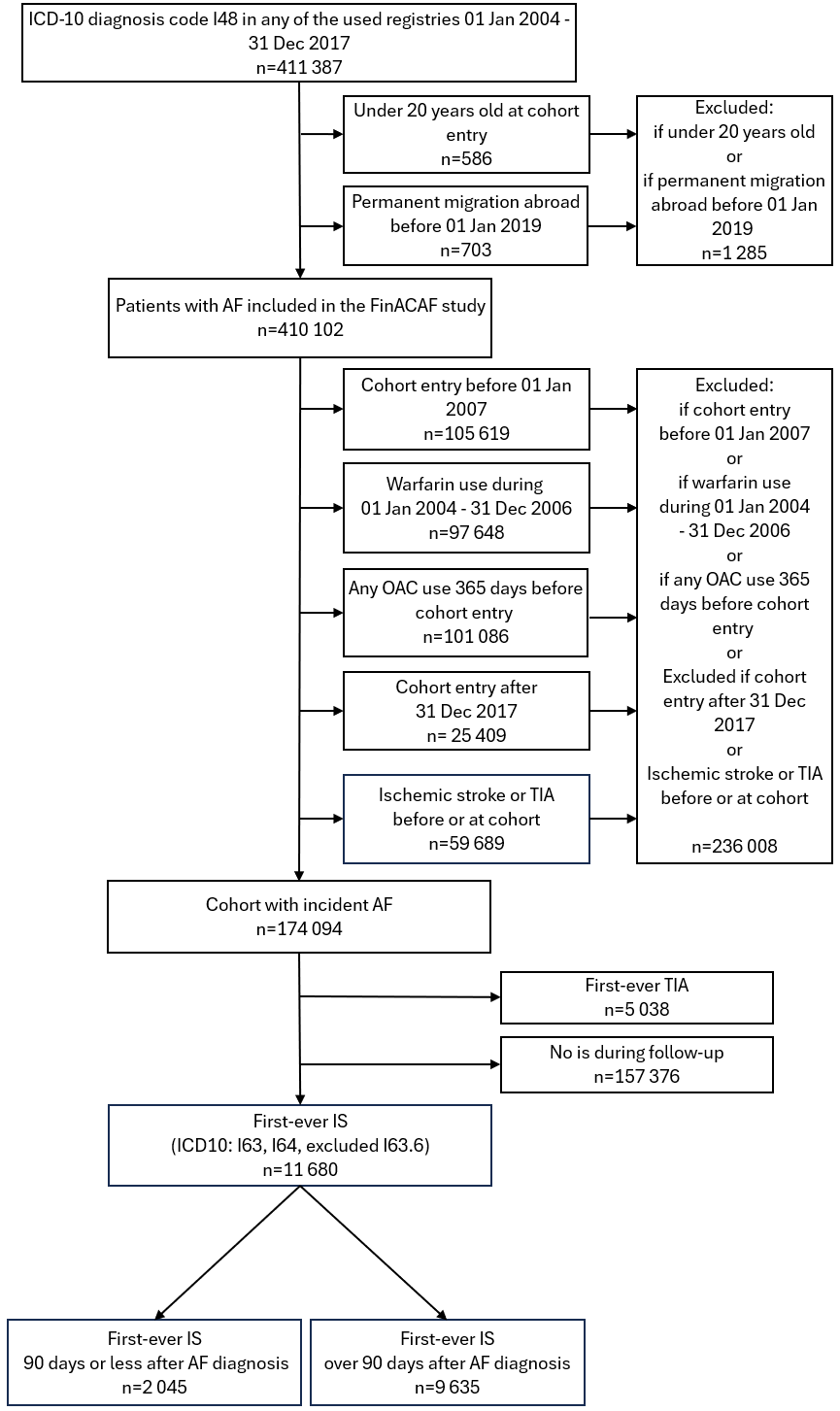
**

**Figure S2. Risk Factors and Interrelations in Ischemic Stroke and Non-OAC Treatment**


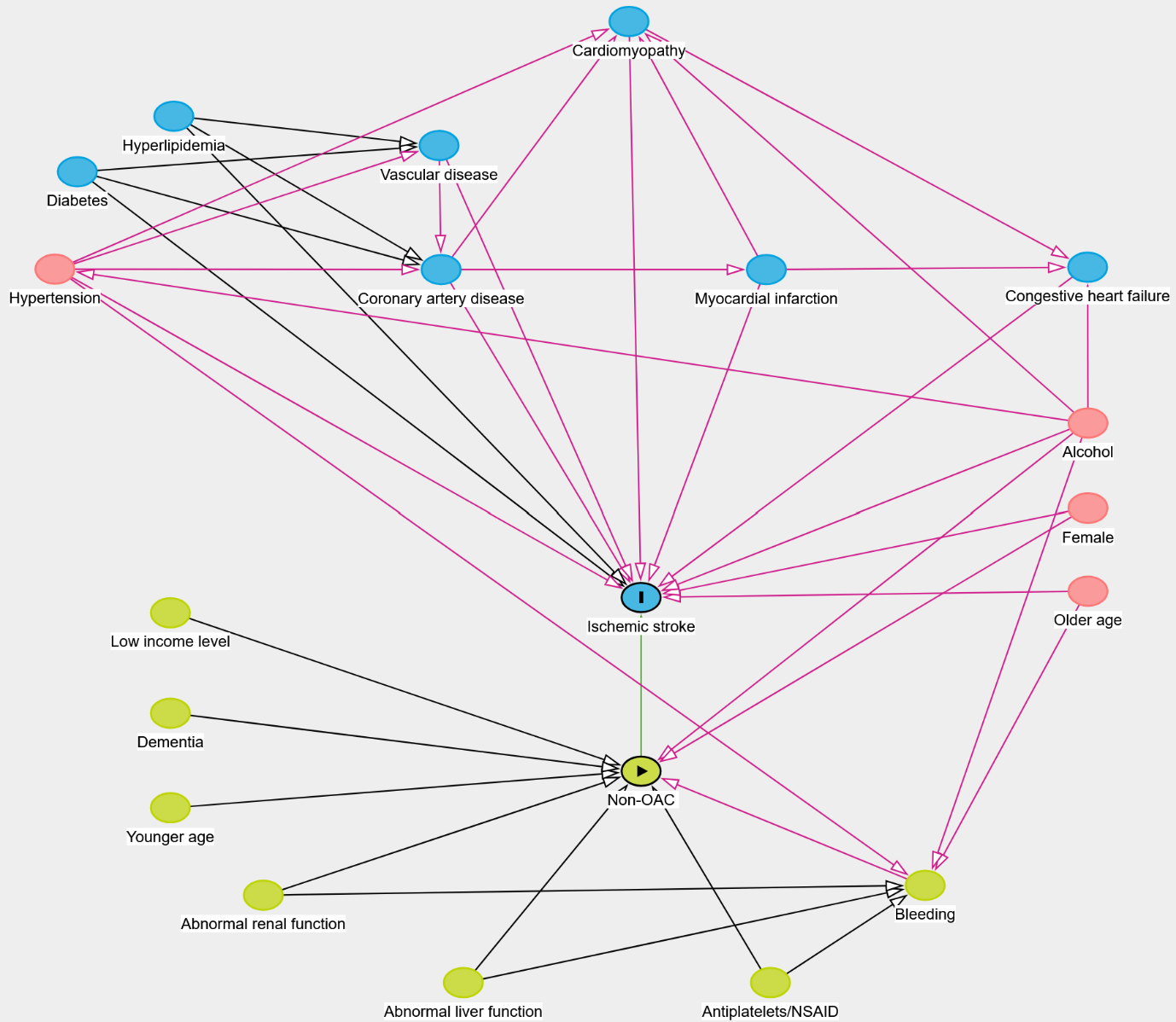


**Figure S3**

Fine-Gray subdistribution hazard model for ischemic stroke (IS) and all-cause death as competing events in patients with atrial fibrillation.

**
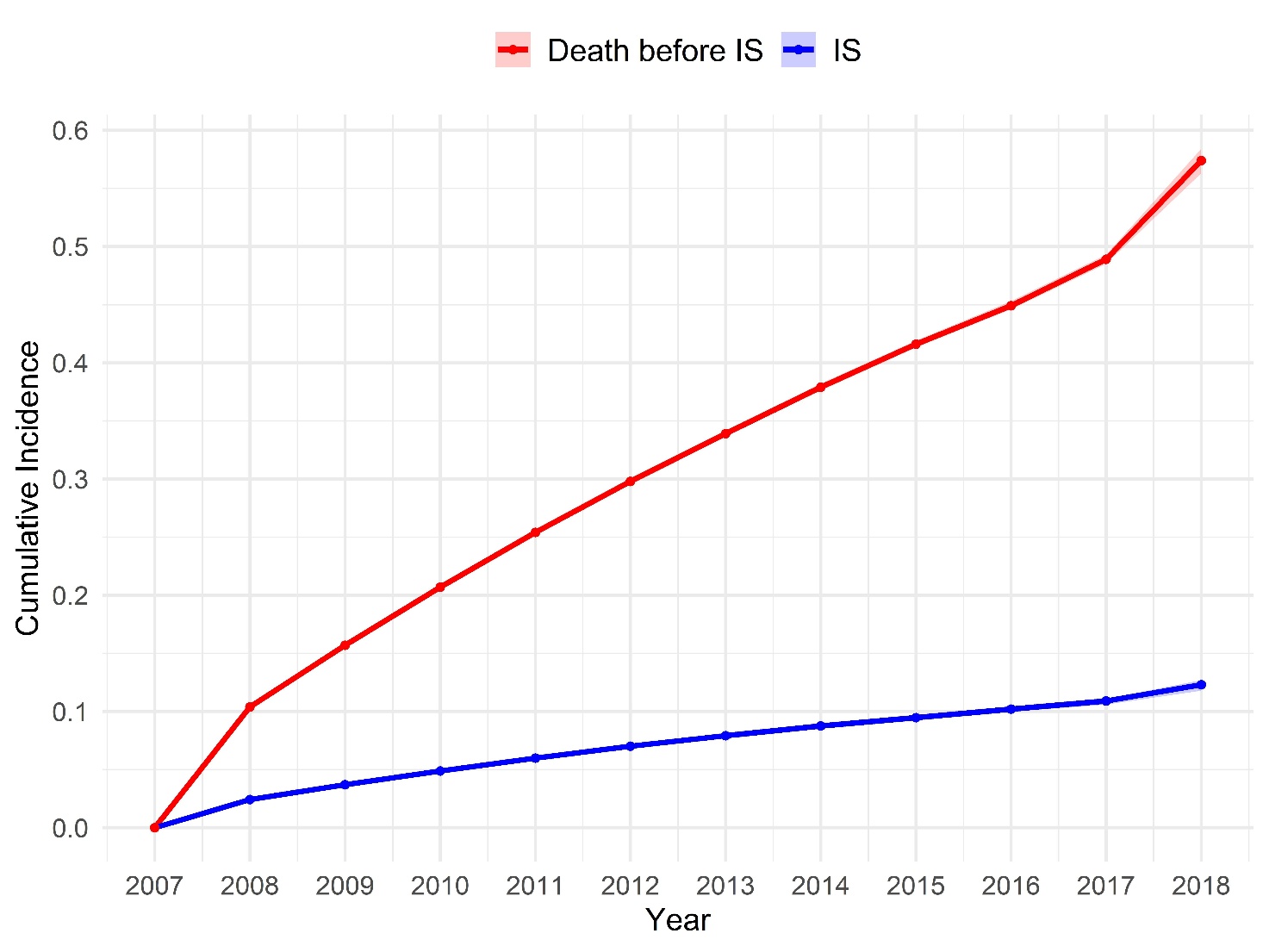
**

**Figure S4 A–B.
A,** Annual proportion of oral anticoagulation purchases in first-ever ischemic stroke patients with atrial fibrillation, when CHA₂DS₂-VA ≥2. Shaded areas denote Clopper-Pearson 95% confidence intervals.
**B,** Annual proportion of oral anticoagulation purchases in atrial fibrillation patients without stroke, when CHA₂DS₂-VA ≥2. Shaded areas denote Clopper-Pearson 95% confidence intervals. DOAC indicates direct oral anticoagulant.


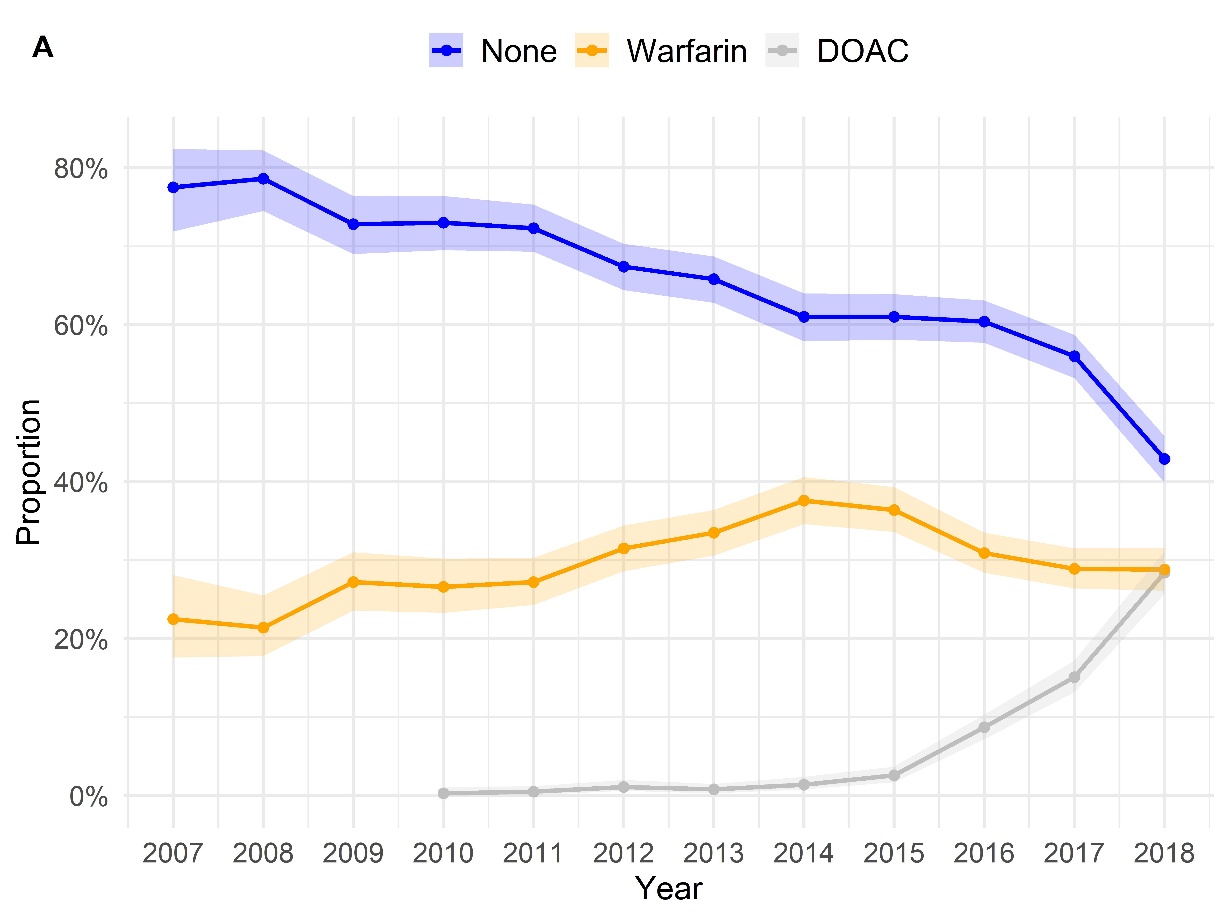


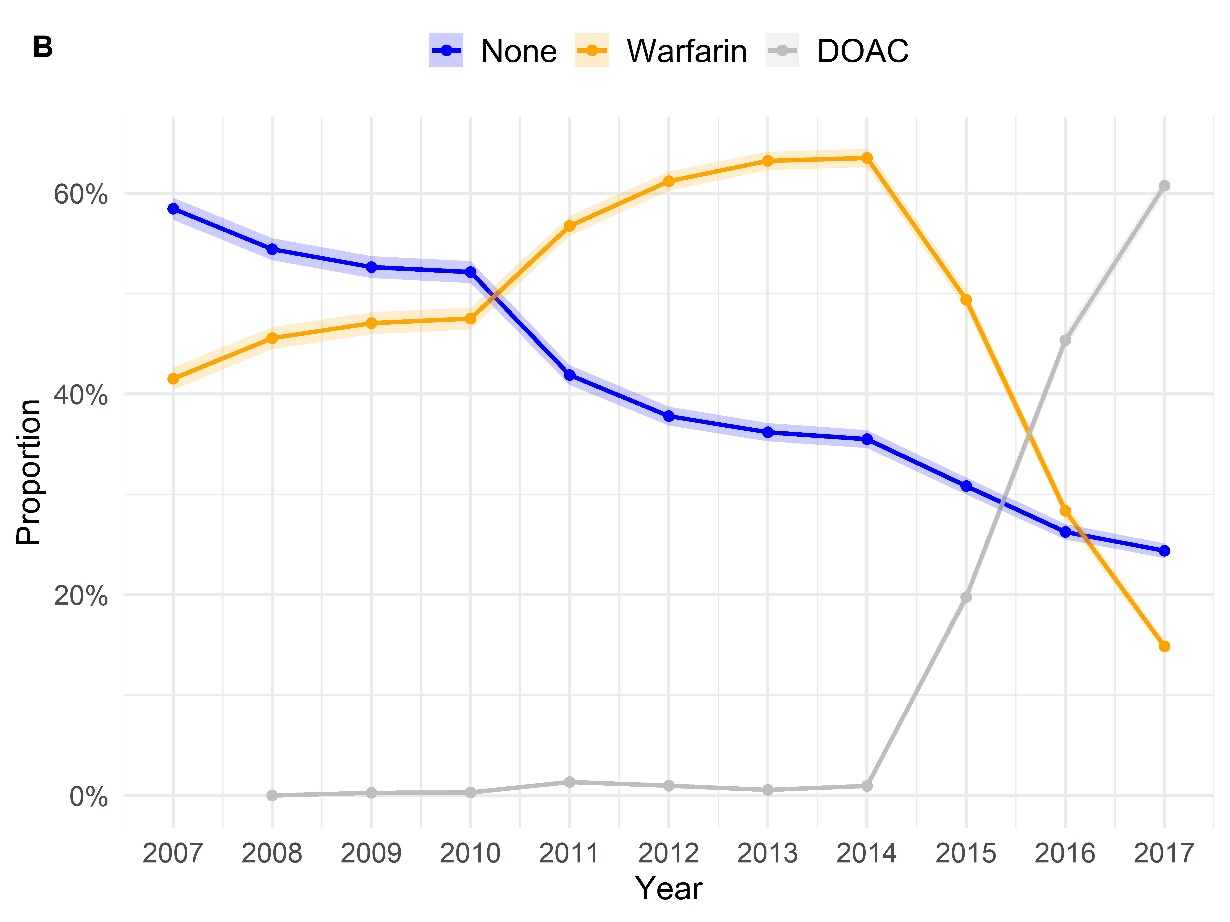

Supplement: sj-docx-1-eso-10.1177_23969873251343857 – Supplemental material for Factors associated with oral anticoagulant non-use at first ischemic stroke in atrial fibrillation: A nationwide study [file sj-docx-1-eso-10.1177_23969873251343857.docx]
