# Supplementary figures and images for: Factors associated with oral anticoagulant non-use at first ischemic stroke in atrial fibrillation: A nationwide study
Source: Eur Stroke J. 2025 Jun 20:23969873251343857. Online ahead of print. doi: 10.1177/23969873251343857 (PMC12182559; doi:10.1177/23969873251343857)

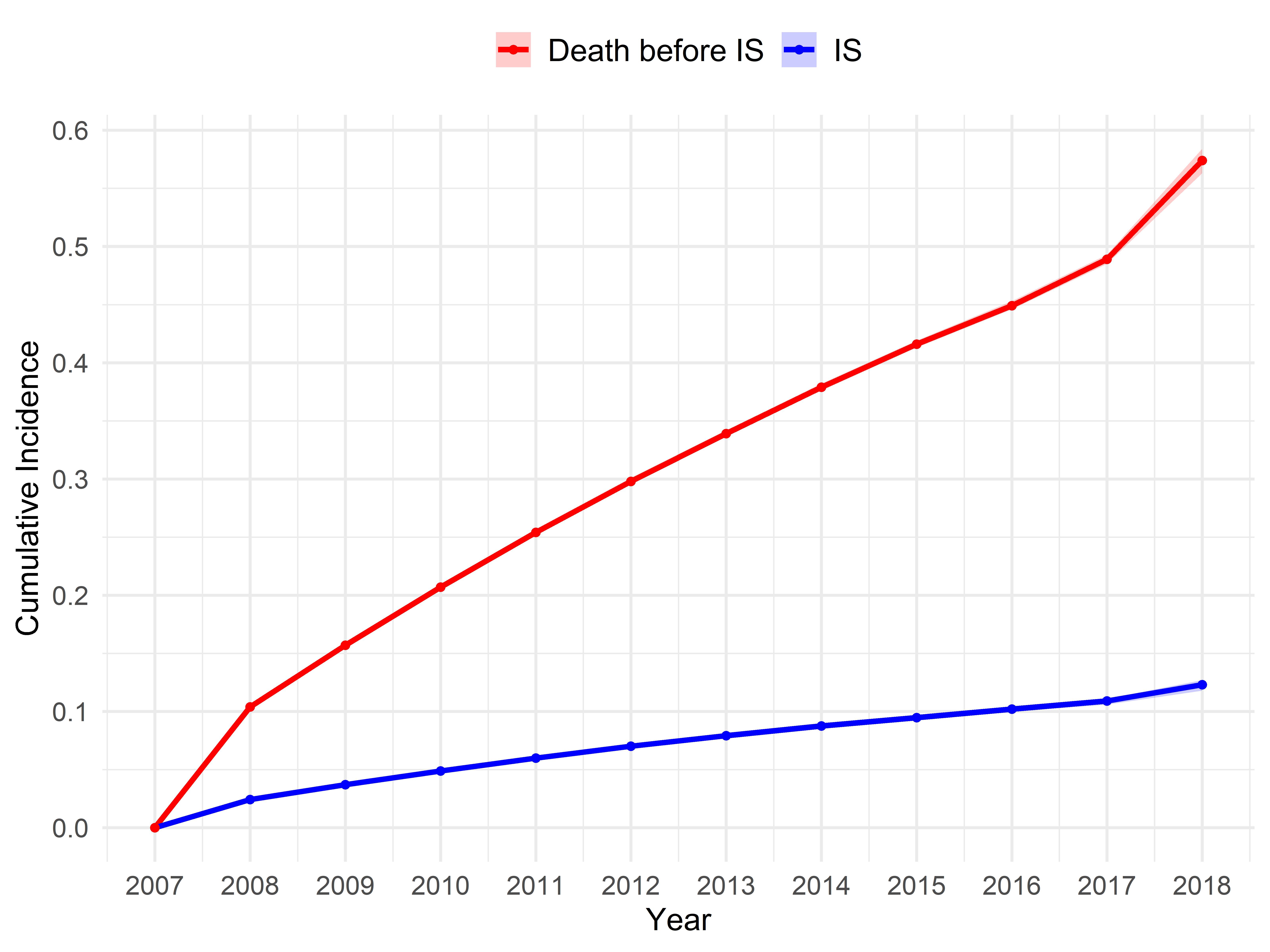

Supplement: sj-jpeg-2-eso-10.1177_23969873251343857 – Supplemental material for Factors associated with oral anticoagulant non-use at first ischemic stroke in atrial fibrillation: A nationwide study [file sj-jpeg-2-eso-10.1177_23969873251343857.jpeg]

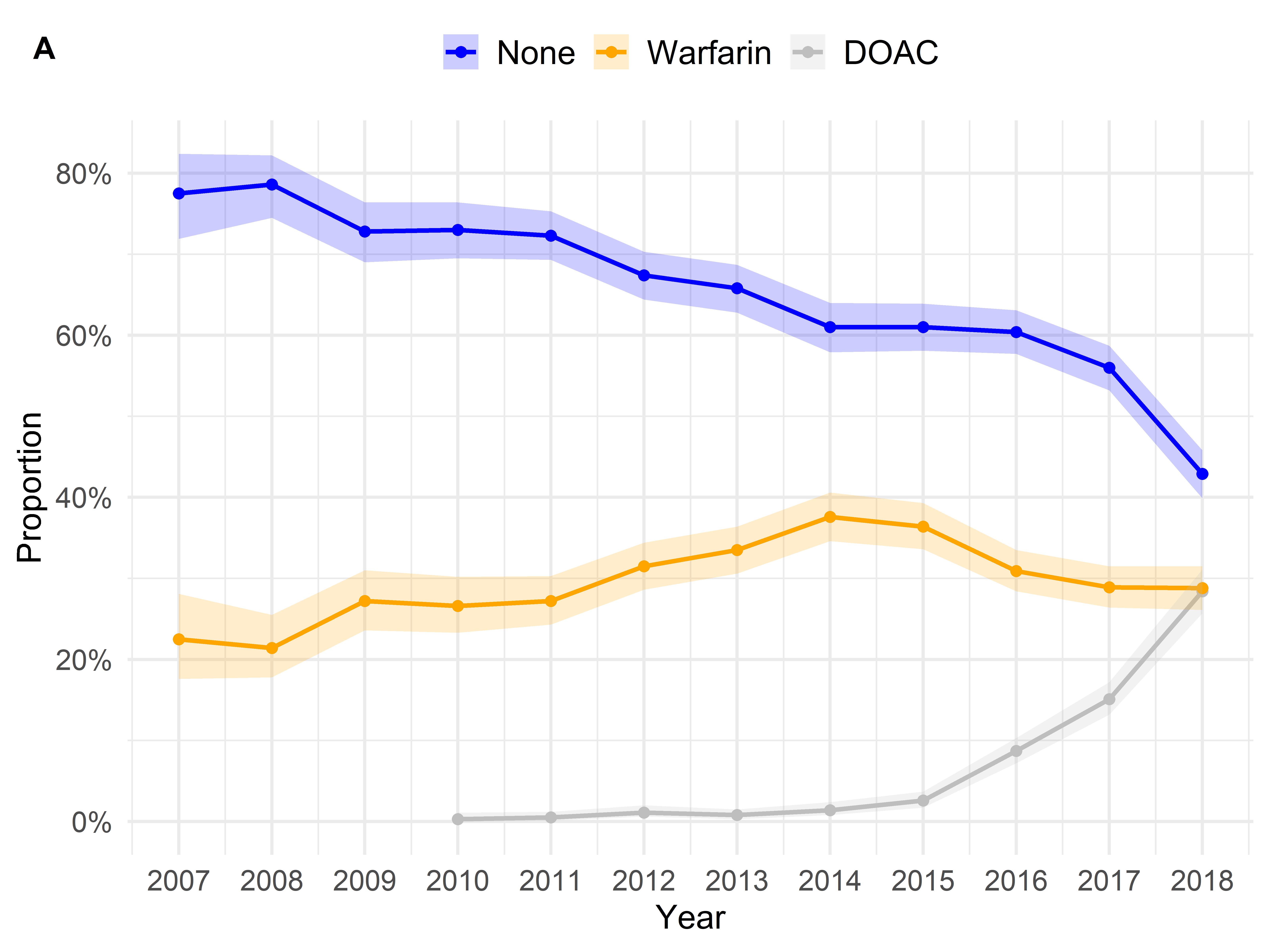

Supplement: sj-jpeg-3-eso-10.1177_23969873251343857 – Supplemental material for Factors associated with oral anticoagulant non-use at first ischemic stroke in atrial fibrillation: A nationwide study [file sj-jpeg-3-eso-10.1177_23969873251343857.jpeg]

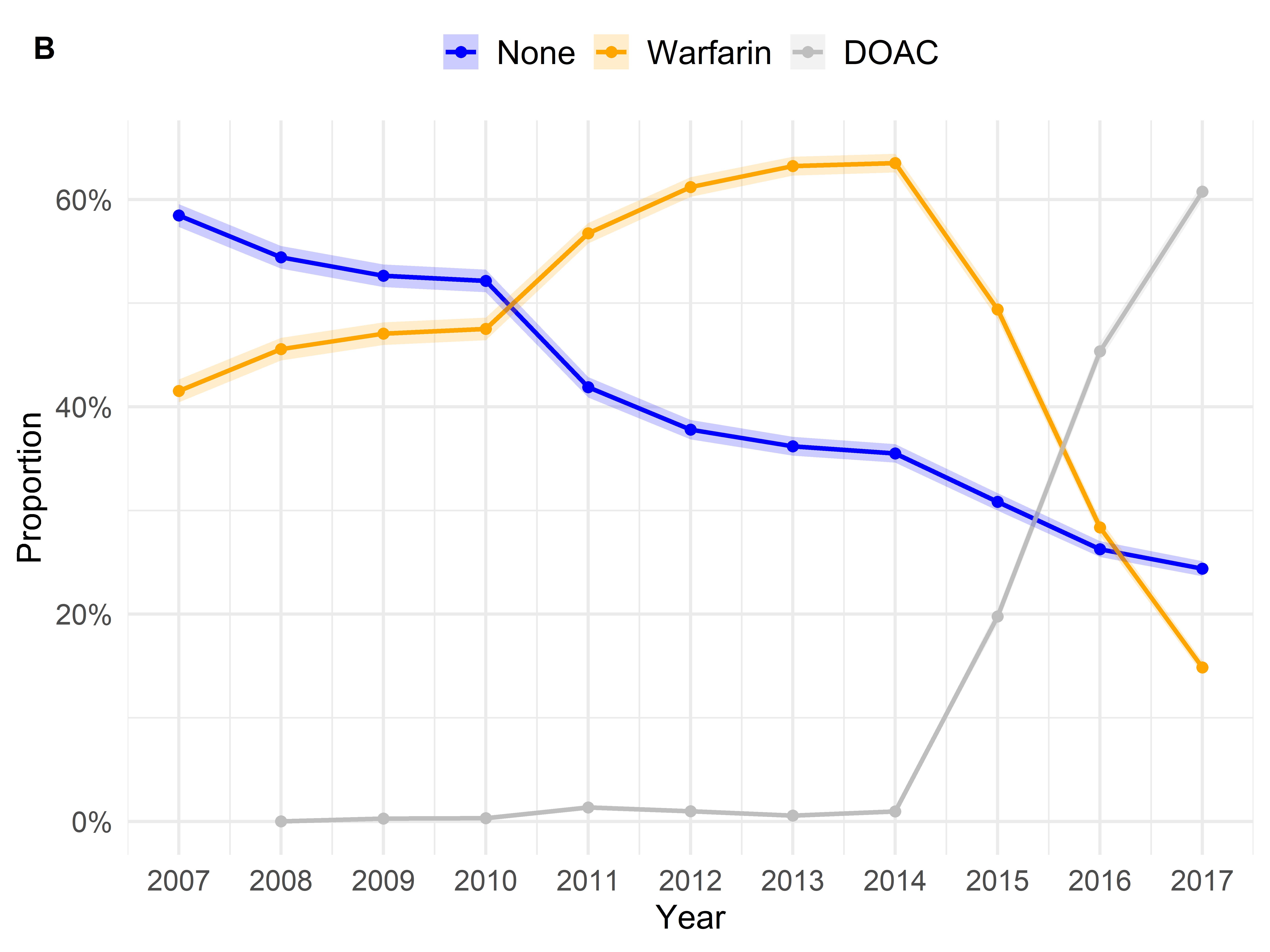

Supplement: sj-jpeg-4-eso-10.1177_23969873251343857 – Supplemental material for Factors associated with oral anticoagulant non-use at first ischemic stroke in atrial fibrillation: A nationwide study [file sj-jpeg-4-eso-10.1177_23969873251343857.jpeg]
